# Toward Precision Medicine in Atherosclerotic Cardiovascular Disease: Insights from Omics Data into Sex Differences

**DOI:** 10.1007/s11883-025-01380-1

**Published:** 2025-12-29

**Authors:** Jelena Munjas, Sandra Vladimirov, Tamara Ratkovic, Laura Comi, Claudia Giglione, Ilija Tanaskovic, Tamara Gojkovic, Branka Rakic, Aleksandar Davidovic, Luka Vukmirovic, Marko Milanov, Dane Cvijanovic, Paolo Magni, Miron Sopic

**Affiliations:** 1 Department of Medical Biochemistry, Faculty of Pharmacy, Faculty of Pharmacy, University of Belgrade, Belgrade, Serbia; 2 Department of Pharmacological and Biomolecular Sciences ‘Rodolfo Paoletti’, Università degli Studi di Milano, Via G. Balzaretti 9, Milano, 20133 Italy; 3 IRCCS MultiMedica, Via Milanese 300, Sesto San Giovanni, 20099 Italy; 4 Institute for Artificial Intelligence, Novi Sad, Serbia; 5 Intern Clinic, Clinical Ward for Cardiovascular Diseases, Clinical-hospital Centre Zvezdara, Belgrade, Serbia; 6 Department for internal medicine, Faculty of Dentistry, University of Belgrade, Belgrade, Serbia

**Keywords:** Omics, Sex differences, Atherosclerotic cardiovascular disease, Precision medicine, Biomarkers

## Abstract

**Purpose of Review:**

Atherosclerotic cardiovascular disease (ASCVD) remains a leading cause of morbidity and mortality worldwide. Although there is increasing recognition of sex differences in ASCVD epidemiology, pathogenesis, and clinical outcomes, the underlying biological mechanisms are still insufficiently understood. Women often present with distinct disease phenotypes, such as a higher prevalence of fibrous plaques and microvascular dysfunction, compared with the lipid-rich, inflammatory plaques more typical in men. This review examines recent omics research to clarify the molecular basis of these sex-specific patterns and explores their implications for precision cardiovascular medicine.

**Recent Findings:**

Advances in genomics, epigenomics, transcriptomics, proteomics, and metabolomics have shown that sex differences in ASCVD arise from complex hormonal, genetic, epigenetic, and molecular interactions. The variety of available omics approaches offers the potential to discover sex-specific regulatory networks and therapeutic targets, thereby addressing persistent knowledge gaps. However, significant challenges remain, including integrating these diverse omics layers, harmonising datasets across platforms, managing substantial computational demands, and navigating ethical constraints related to data sharing.

**Summary:**

Multiomics technologies provide unprecedented opportunities to dissect sex-specific mechanisms in ASCVD and to refine individualised risk stratification and therapeutic strategies. Overcoming current analytical and infrastructural barriers through collaborative efforts, standardised methodologies, and responsible data governance will be critical to unlocking the full potential of multiomics in precision cardiovascular medicine. This review synthesises recent evidence across omics domains and underscores their potential to improve ASCVD prevention and treatment.

**Graphical Abstract:**

Graphical abstract. Illustration of omics layers — genomics, epigenetics, transcriptomics, proteomics, metabolomics — and their role in uncovering sex-specific mechanisms that contribute to ASCVD. Image generated with BioRender (biorender.com).

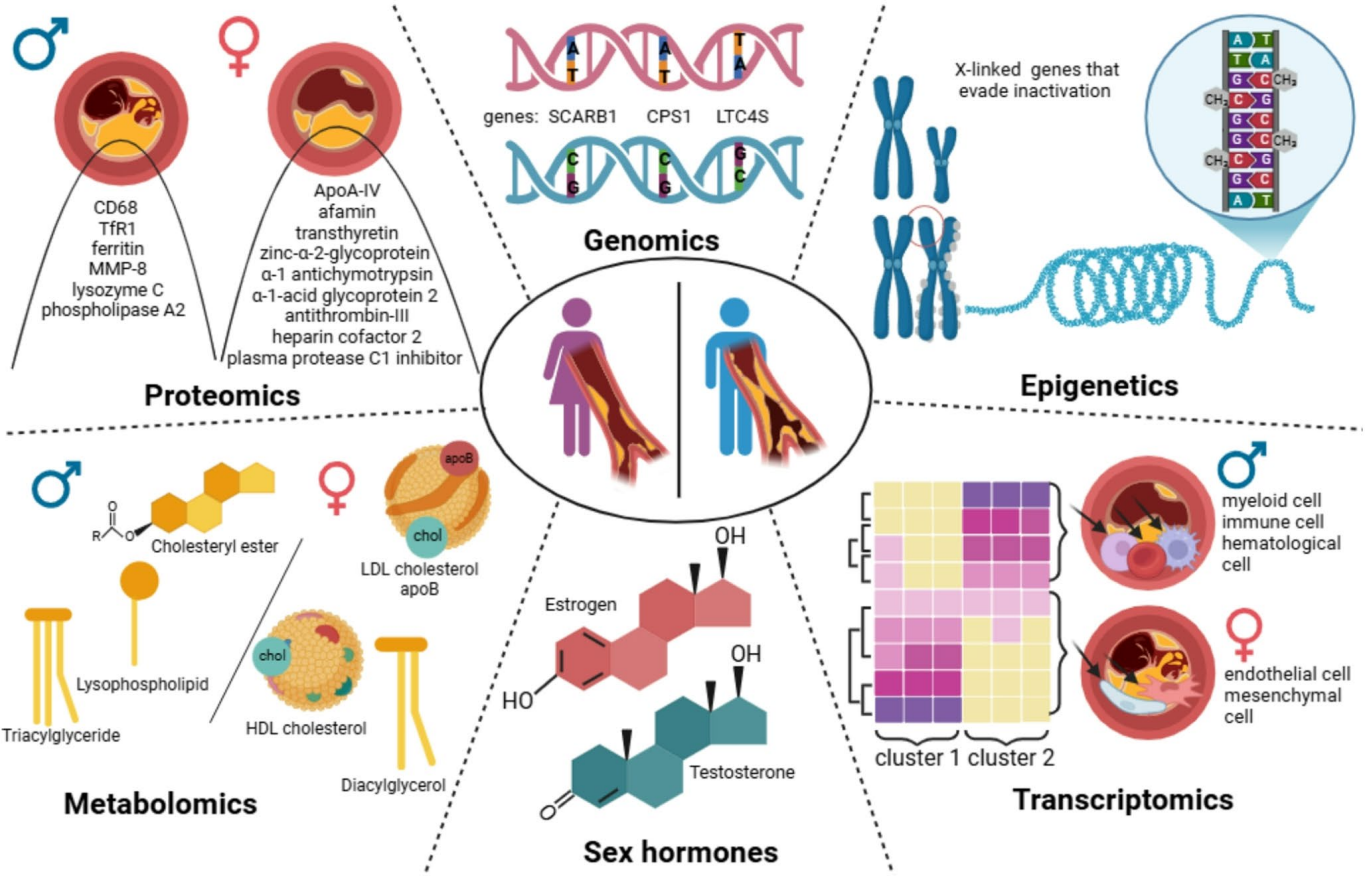

## Introduction

According to the Global Burden of Cardiovascular Disease, 9.6 million men and 8.9 million women globally died from cardiovascular disease (CVD) in 2019 [1]. More importantly, atherosclerotic CVD (ASCVD) mortality is increasing, with the fastest relative increase in middle-aged women. ASCVD exhibits notable differences in epidemiology and pathogenetic mechanisms between men and women, and their understanding is crucial for accurate risk assessment and effective targeted prevention strategies. Sex differences in ASCVD disease presentation, prognosis and associated risk factors have been widely studied [2–4]. Acute myocardial infarction (AMI) remains a leading cause of death in women [5]. Obstructive atherosclerotic disease of the epicardial coronary arteries is still the basic cause of AMI in both sexes. However, plaque characteristics differ, with women having increased prevalence of fatalities due to plaque erosion [6], while in men plaque rupture is a more frequent cause. Men also tend to develop atheromatous, lipid-rich plaques, with more inflammatory cells, calcification, lipids and hemorrhage, while women are more likely to develop fibrous plaques with high collagen and smooth muscle cell (SMC) content, accompanied with endothelial dysfunction [7]. Recent data have suggested a greater role of microvascular disease in the pathophysiology of coronary events among women [3, 6]. Serum calcification propensity, mirroring vascular calcification, is associated with increased mortality in patients with CVD. It is increased in AMI patients with ST-segment elevation (STEMI), compared with the general population, and its contribution is more pronounced in women than in men [8]. Women paradoxically have less severe obstructive disease of their epicardial coronary arteries at elective angiography than men [6]. Moreover, women more commonly present with AMI without ST-segment elevation (NSTEMI), nonobstructive coronary artery disease (CAD), and more likely have unusual pathophysiological mechanisms of CAD (such as spontaneous coronary artery dissection) [9].

Sex differences are depicted in different clinical presentation, resulting in detrimental consequences for diagnosis and treatment, and subsequently worse outcomes and increased rates of readmission, reinfarction, and death in women [10]. Multiple studies have shown that women with acute coronary syndromes (ACS) are less likely to be treated with guideline-directed medical therapies, to undergo cardiac catheterization, and to receive timely reperfusion [6]. Regardless of age, major adverse cardiovascular events (MACE) that occur 1–5 years after AMI such as re-infarction, death, development of heart failure and stroke, are significantly more frequent in women than in men [6]. Responsibility for recurrent events after AMI is often attributed to native, non-flow limiting, non-culprit lesions. It was shown that female patients’ lesions harboured more high-risk plaque features compared to male [11].

ASCVD risk factors incidence and effects considerably differ between sexes [12]. Although more men smoke than women [13], smoking seems to impact to a greater extent in women, since it increases the risk of coronary artery disease (CAD) by 25% [14] and myocardial infarction (MI) by over 50% in women compared to men [15]. Gene expression data of 625 carotid plaques from current smokers show sex-dependent upregulation of SMC gene *CRLF1*, which may explain the different contributions of smoking to CVD risk in females [16]. Type 2 diabetes mellitus (T2DM) is more prevalent in men than in women (14.6% vs. 9.1%), but women with T2DM face a threefold higher CVD mortality risk compared to a doubled risk in men. Women with T2DM also experience faster deterioration in CVD risk factors, including blood pressure and lipid profiles, leading to higher mortality rates [12]. Adverse changes in weight, lipids, blood pressure, and glucose metabolism with menopause transition highlight potential accelerating CVD risk [17].

Biological sex-based differences between females and males comprise genetic differences and differences in hormonal status. At variance, sociocultural gender refers to socially constructed norms that impose and determine roles, relationships and positional power for individuals in a specific society and time [18]. Sex-related and gender-related factors either have synergistic or opposing effects on the clinical manifestations and outcomes of CVD [18]. Female-specific, gender-related risk factors, such as pregnancy-associated disorders, should be considered to promote earlier ASCVD risk factor assessment [17]. Other female gender specific factors including breast cancer therapy, autoimmune and rheumatic diseases, depression, and household-related stress, significantly impair outcomes in women with CAD or heart failure compared with those in men [18]. In this review, we will focus on biological, sex-differences between females and males.

Missed or delayed diagnosis and undertreatment do not fully explain this burden of ASCVD in women. Sex-specific mechanisms underlying atherosclerosis are multifactorial [17], and their understanding is crucial for developing targeted prevention and treatment strategies for atherosclerosis in men and women.

In this review, we highlight specific genetic, epigenetic, transcriptomic, proteomic and metabolomic features of sex-differences in atherosclerosis and discus the potential of multiomic data integration in understanding sex differences in atherosclerosis and improving ASCVD management in women.

### Genomics

Since the Human Genome-Wide Association Studies (GWAS) era has taken progress in 2007, 321 gene loci linked to CAD [19], and hundreds of candidate genes involved in atherosclerotic pathophysiology have been identified [7]. In addition, the risk for developing ASCVD is also strongly influenced by sexual dimorphism correlated with X and Y chromosomes. The presence of Y chromosome and the dosage of X chromosome both lead to sex-dependent transcriptional differences and sexual dimorphism [20]. According to the differential expression of X/Y chromosomal genes, several sex-specific CVD phenotypes exist, each potentially having a different impact on CVD risk [20]. A large-scale genome-wide association study of CAD, which included over a quarter of a million cases, identified 9 novel loci on the X chromosome [21]. Sex-stratified GWAS in a subset of 77,080 CAD cases (originating from another large scale study with over a million participants) found ten associations that reached genome-wide significance and had evidence for between-sex heterogeneity. Nine of the 10 had stronger effects in the male participants, and only the MYOZ2 (rs7696877) locus had a significantly larger effect in females. This finding could be a reflection of the larger sample size for males [22]. By analyzing data from the open access UK Biobank, a Y chromosome haplogroup associated with higher CVD risk was identified, influencing proatherosclerotic reprogramming of the transcriptome [23]. In another study, conducted in British men, several haplotypes of the Y chromosome were associated with a 50% increase of age-adjusted risk for CAD [24]. Ninety variants on X chromosome, and single nucleoid polymorphisms (SNPs) in autosomal genes were found to be significantly associated with nonobstructive CAD in women [25]. A GWAS, sex-stratified study, identified SNPs in *SCARB1 (*scavenger receptor class B, member 1), a plasma membrane receptor for HDL, and in carbamoyl-phosphate synthase 1 (*CPS1*), associated with CAD in women but not in men [26]. GWAS studies based on carotid intima-media thickness (CIMT), an early marker of atherosclerosis, identified SNP in *LTC4S* gene (leukotriene C4 synthase) associated with CIMT in women, but not in men [27]. In addition, SNP-by-sex correlation for *LEKR1* (leucine, glutamate and lysine rich 1) and (polypeptide N-acetylgalactosaminyltransferase 10), whose loci are associated with adiposity and weight control, was observed [28]. The analysis of SNPs linked to atherosclerotic risk factors and other CVD risk factors also showed positive correlation of SNPs in *LPA* (lipoprotein (a)), *MYBPH* (myosin binding protein H), *ADORA3*, (adenosine A3 receptor) and *PON* (paraoxonase 1) genes mainly in women compared to men [26]. Despite GWAS approach, it has been challenging to characterize CVD risk according to the role of genetic sex (e.g. XX and XY chromosomes), especially if variables like gonad type, gender or high prevalence of congenital CVD abnormalities and hypogonadism are considered. Moreover, CVD risk is influenced by the gain or the loss of sex chromosomes. For instance, men with Klinefelter syndrome carrying an extra copy of the X chromosome (XXY genotype), have an increased CVD risk [29]. GWAS with larger sample sizes particularly on female CAD cases would be crucial in future for a better understanding of the sex-specific genetic basics of CAD [19]. In addition, biological significance of sex chromosomes has been comprehensively described elsewhere [30–32].

Analyses in the UK Biobank showed that CAD polygenic risk score (PRS), which can predict and improve CAD risk discrimination, performs worse in women relative to men. GPSmult, a multiancestry CAD PRS, was developed from data from the Mass General Brigham Biobank, and sex-specific analysis were used to create a sex‐differential polygenic risk score, PRSsexdiff. A linear combination of GPSmult, PRSsexdiff, and the base model of age, batch, and first 5 principal components of ancestry yielded improved, but not fully resolved, CAD risk association effect in female participants. But it should be noticed that CAD association was heightened for women aged < 55 years, emphasizing greater utility identifying actionable early risk [33].

### Epigenetics

Epigenetics examines heritable changes in gene function that do not involve alterations to the DNA sequence itself. Epigenetic mechanisms typically encompass DNA methylation, histone modification, and the regulatory effects of non-coding RNAs (ncRNAs). In this section, we focus specifically on DNA and histone modifications in the context of sex differences in ASCVD, while the role of ncRNAs in this process is explored in the following section. Epigenetic modifications involve tissue-specific and dynamic changes within the genome that influence gene expression, maintain chromosomal stability, and regulate genomic imprinting. These modifications allow cells to adapt gene activity to specific needs, reflecting environmental and developmental signals, without altering the DNA sequence [34, 35]. DNA methylation represents the most extensively studied epigenetic mechanism in CVD. It plays a crucial role in X-inactivation and genomic imprinting, modulating the effects of risk factors involved in CVD [7].

The XY sex chromosome pair in males versus the XX sex chromosome pair in females represents the most prominent differences in genetic material between sexes. Inactivation of one of the two X chromosomes in females is orchestrated by DNA methylation and represents a mechanism that ensures that the same amount of genes is present in both males and females. However, around 20% of X-linked protein coding genes may evade inactivation, remaining active, overexpressed in women, representing important targets for studying sex differences in CVD. X-inactivation is a tissue-specific process which contributes to differences seen in cardiovascular phenotypes between sexes [30, 36–38]. Sex-biased gene expression was observed in 22 genes that escape X chromosome inactivation [39]. The sex-biased genes are enriched for proteins that are involved in deposition of epigenetic marks (such as histone modifications) and for genes that are adjacent to a subset of transcription recognition sequences, including hormone-related transcription factors [39]. For instance, genes coding two histone demethylases (KDM5C and KDM6A), which modify methyl marks on histone tails resulting in the regulation of several sex-biased genes, escape X inactivation [40]. More gene loci, including DNA demethylase; Eif2s3x gene, involved in translation initiation; and RNA helicase Ddx3x, involved in transcriptional regulation, pre-mRNA splicing, mRNA export, cellular signalling, and viral replication, are all located on the X chromosomes and evade inactivation [36, 37].

DNA methylation contributes to the differential expression of sex-biased genes linked with CVD risk factors. DNA methylation patterns at 31 gene sites were associated with sex differences in blood lipid levels, stroke risk, and other cardiometabolic traits. Prioritized pathway connectivity analysis associated these genes with vitamin B12, cholesterol and plasma lipoprotein metabolism, plasma lipoprotein assembly and remodelling and statin pathway [34]. Six regions (two within known CAD loci) showed sex-specific methylation in blood of CAD patients [7], which were related to sex-specific effects in lipid metabolism, endothelial dysfunction and vascular remodelling, without involvement in estrogen signalling pathways. However, these were candidate gene studies from blood samples, focused on the methylation of preselected genes without mentioning the identified CpG site. On the other hand, epigenome wide approach from atherosclerosis plaques provides a more effective understanding of atherosclerosis process itself. Genome-wide DNA methylation and transcriptomics analysis on plaques of 485 carotid endarterectomy patients, identified 4848 autosomal CpG sites, most of which were hypermethylated in female atherosclerotic tissue compared to male tissue. This considerable amount of sex-differential CpGs might represent the known sex [35] differences in plaque biology between men and women [7, 35]. A recent study by Benavente et al. showed that atherosclerotic plaque epigenetic age acceleration (EAA) is a strong and independent marker of a poor outcome in both male and female patients with severe atherosclerosis, but also found a significant sex difference in plaque epigenetic age of around 2 years, which is perhaps too short to explain the observed sex differences in plaque phenotype and outcome. Authors argue that it could either be a consequence of biological difference or driven by the Horvath clock, used to estimate EEA, by which epigenetic age in females is lower as compared with males. On the other hand, it could be due to the fact that females develop atherosclerosis-related complications a decade later than men [41].

Additionally, sex-biased epigenetic mechanisms have been reviewed in detail elsewhere [7, 34, 42].

### Transcriptomics

Transcriptomics offers comprehensive insights into RNA, capturing the genome’s functional and dynamic output. In the context of sex differences, transcriptomics provides valuable insights into how genes are actively transcribed into RNA, allowing researchers to observe distinct patterns in cellular responses, development, and disease mechanisms between sexes. For example, transcriptomic studies across 44 human tissues, over > 37% of the entire human transcriptome is differentially expressed among males and females in at least one tissue [39]. Most of the sex-biased genes involve autosomal genes, whereas only 4% are related to X chromosome [39, 40]. Recent transcriptomics and single-cell RNA sequencing analysis of the human atherosclerotic plaques have opened the path for a more profound understanding of sex-specific features of ASCVD.

Transcriptomics and single-cell RNA sequencing data from atherosclerotic plaques revealed sex differences in SMC and endothelial cell biology, highlighting that phenotypic switching of smooth muscle cells plays a crucial role in female atherosclerosis. Namely, single-cell RNA sequencing of fibrous atherosclerotic plaques revealed that female-biased genes are mostly expressed in SMCs, and higher expressed in SMCs from female (predominantly stable) plaques as compared to male (relatively unstable) plaques [35]. Female-biased genes were enriched for protease inhibitors, such as *TIMP2* and *TIMP3*, proteins that can potentially halt the remodelling leading to unstable plaques, while ribosomal protein enrichment points to enhanced protein turn-over. Male SMC genes were enriched for immediate early response transcription factors, such as *JUNB* and *FOSB*, as well as responses to lipids and cytokines. Genes which encode the extracellular matrix proteins (*COL3A1*,* TIMP2*,* TIMP3*,* ASPN*) showed sex difference in expression in SMCs within the plaque.

Analysis of differentially expressed genes (DEGs) in human aortic SMCs under a proliferative state (induced by growth factor stimulation) and a combined state (induced by simultaneous exposure to growth and inflammatory stimuli) revealed sex-specific transcriptional responses to these activation conditions. In the proliferative state, DLL4 and NOTCH4, key regulators of vascular development and remodeling, were upregulated in male SMCs. In contrast, under the combined condition, sex-associated DEGs such as EIF1AXP1, DKK2, GALNT13, and SPARCL1 were identified, suggesting activation of distinct molecular pathways whose roles in sex-biased atherosclerosis warrant further investigation [43].

Studies of complex genetic regulatory networks (GNRs) have pointed to significant differences in key genes involved in the atherosclerosis process in women and men, suggesting that the basis of disease progression in men and women is different [3]. Connectivity patterns within the atherosclerotic tissues indicated the existence of two gene clusters with clear sex differences [3]. The first gene cluster, in which a better connection between genes was shown in male atherosclerotic tissue, included genes mainly expressed in myeloid, immune, and hematological cells. On the other hand, the second cluster, in which a better connection between genes was shown in atherosclerotic tissue from females, included genes mainly expressed in endothelial and mesenchymal cells [3].

Furthermore, growth arrest-specific protein 6 (GAS6) and serpin family G member 1 (SERPING1), two genes identified as key drivers of gene regulatory networks active in females with CAD, were found to be dominantly expressed in phenotypically altered plaque’s SMCs [3]. In particular, Hartman et al. have shown that the expression of GAS6 is regulated by the transcription factor klf4, an important regulator of SMC phenotype switch. This could indicate that the transformation of plaque’s SMC characteristics is influenced by sex differences [3]. The GAS-6-Axl receptor tyrosine kinase (GAS6-Axl) signalling pathway has a role in downstream activation of the phosphatidylinositol 3-kinase-protein kinase B (PI3K). PI3K activates Akt1, which has been shown to contribute to plaque stabilization by numerous mechanisms, including the reduction of SMC apoptosis [3, 44]. In this way, the activation of GAS6 may affect the stabilization of the plaque. Since it has been shown that estrogen, through estrogen receptors (ER) on endothelial cells, affects the expression of GAS6, it might similarly play a role in GAS6 activity in plaque SMCs. Considering that GAS6 expression is higher in plaques in women compared to men, as well as in stable plaques compared to unstable, it can be presumed that ER-mediated signals may contribute to sex-specific mechanisms of plaque stabilization [3]. These data indicated relevance of GNR driven by GAS6 in SMC differentiation and suggested that sex differences in plaque biology involved phenotypic switching of plaque SMCs. In addition, two recently identified SMC GRNs in female carotid plaques strongly overlap with this GRN [45].

Additionally, integration of deep single-cell RNA sequencing of human carotid plaques with GRNs derived from the multi-omics Stockholm–Tartu Atherosclerosis Reverse Network Engineering Task (STARNET) study revealed distinct subcellular clusters in male and female plaques, reflecting differences in plaque phenotypes. These sex-specific GRNs provide mechanistic insight into the molecular basis of sex-biased atherosclerotic cell states. Female plaques were characterized by GRN33 and GRN122, associated with lipid-laden inflammatory macrophages and endothelial–mesenchymal transition, whereas male plaques were enriched for GRN195, linked to angiogenesis and T-cell–mediated inflammation [46].

Genes mainly related to inflammatory pathways, such as the Janus kinase/signal transducers and activators of transcription (JAK/STAT) signalling pathway, are more prominently expressed in vulnerable plaques in men, in contrast to the profibrotic epidermal growth factor receptor (EGFR) and transforming growth factor beta (TGFβ) pathways, which are more prominent in stable plaques in women [47]. This gene expression pattern suggests greater plaque stability in women and a more inflammatory plaque milieu in men [47].

It is worth mentioning that the largest part of the transcriptome is attributed to non-coding RNA species. Among them, microRNAs are the most studied so far in human diseases. They represent small, about 22–24 nucleotides long, noncoding RNAs that regulate gene expression at the post transcriptional level [48]. A growing body of evidence supports the involvement of miRNAs in ASCVD pathogenesis, but there is limited information regarding their sex-specific involvement in ASCVD. In addition, emerging evidence suggests that alongside miRNAs, long non-coding RNAs (lncRNAs) may play a substantial role in the initiation and progression of atherosclerosis, but also with limited information regarding their sex-specific influence [49]. Interestingly, the X-chromosome encodes more microRNAs than most of the autosomes. According to miRBase microRNA archive [50], the X chromosome encodes 120 microRNAs, whereas the Y chromosome encodes only 6. This discrepancy could be responsible for sex disparities in physiological and disease conditions via different biological processes such as cell death, inflammation, angiogenesis, cardiomyocyte hypertrophy, and fibrosis [51]. Furthermore, microRNAs located on the X chromosome can be modulated by sexual hormones and can also evade X-inactivation [36, 51]. On the other hand, lncRNA Xist, located within the X-linked minimal genetic region, is one of the master-regulators of the complete process of X-chromosome inactivation. It was shown that lncRNA Xist is linked to atherosclerosis, cardiac hypertrophy and fibrosis and, as such, could contribute to the observed sex disparities in CVD [52].

Sex differences in the expression of miRNAs and lncRNAs encoded both by X chromosome and autosomes, have been described in conditions related to atherosclerosis, such as stroke, MI and heart failure [53], with a potential of exploiting these markers for diagnostic and therapeutic purposes. It was recently shown that 57 miRNAs are differentially expressed between males and females in epicardial adipose tissue (EAT) of CAD patients [54], whose quantity, composition and function influences atherosclerosis development [55]. MiR-21 and miR-92a, which play distinct roles in lipid metabolism and inflammation show sex-dependent expression patterns [56]. In addition, silencing of miRNA-144 in male mice enhanced reverse cholesterol transport and promoted the regression of atherosclerosis, whereas in female mice, these effects were much smaller or absent. Part of these effects was mediated by CYP7B. Cheng et al. have further shown that the expression of CYP7B in men’s carotid arteries was significantly higher in patients with stable plaques compared to those with unstable plaques, while in women this difference was not observed. However, the expression of miR-144 did not differ between sexes and was significantly lower in stable plaques. This may suggest that the observed sex differences are due to the differing effects of miRNA-144 in men and women, rather than differences in its expression [57].

In light of the foregoing, data regarding sex-specific involvement of miRNAs and lncRNAs in ASCVD are limited, implying that more studies, employing high-throughput approaches, and sample sizes with enough statistical power, would be needed in order to fully elucidate their sex-specific contribution to the disease.

### Proteomics

Proteomic analysis of atherosclerotic plaques in men and women revealed certain variations that could contribute to differences between genders in plaque stability and inflammation [58]. Increased levels of inflammatory proteins, such as phospholipase A2 and lysozyme C, were observed in men, while acute-phase proteins like alpha-1 antichymotrypsin and alpha-1-acid glycoprotein 2 were more prominent in women. This ratio between inflammatory proteins and acute-phase proteins, which have anti-inflammatory roles, could explain the lower plaque stability in men [58]. Additionally, lower plaque stability in men can be attributed to the increased concentration of matrix metalloproteinase-8 (MMP-8) compared with women. MMP-8 is an enzyme that contributes to plaque destabilization through cell migration, fibrous cap degradation, remodelling, and neovascularization processes [59]. It was also shown that apolipoprotein A-IV (ApoA-IV) and three transport proteins—afamin, zinc-alpha-2-glycoprotein and transthyretin are more abundant in atherosclerotic plaque in women. Afamin is believed to be negatively correlated with inflammatory factors, while zinc-alpha-2-glycoprotein acts as an anti-inflammatory mediator [58, 59]. The higher amount of transthyretin associated with higher amount of apolipoprotein A-IV (ApoA-IV) in the plaques in women may indicate the existence of differences in reverse cholesterol transport between the sexes [60]. Transthyretin, which is bound to HDL, by cleaving ApoA-I, reduces cholesterol efflux, while on the other hand, ApoA-IV is involved in the uptake and metabolism of cholesterol [61, 62]. Proteins that are part of the coagulation cascade, antithrombin-III, plasma protease C1 inhibitor and heparin cofactor 2 are also more abundant in women [58]. The difference in concentration of these proteins, which have an inhibitory effect on coagulation, could potentially contribute to stabilization of plaque, through inhibition of thrombus formation [60].

The increased prevalence of the pro-inflammatory phenotype of macrophages, CD68 in atherosclerosis plaques and certain proteins involved in iron metabolism, are considered as factors for increased inflammation in men vs. women. In terms of the mentioned iron metabolism, concentrations of transferrin receptor 1 (TfR1) and ferritin are more abundant in plaques in men. TfR1 on macrophages may contribute to an increased iron uptake, and a resulting intracellular concentration of iron, in the form of ferritin. This increase in iron concentration initiates iron-mediated oxidative processes and increases oxidative stress which is in close correlation with inflammation in atherosclerotic lesions [63].

In a recent study, it was shown that in addition to sex-differences in the proteomic profiles of plaques related to inflammation and calcification, there are differences in clusters containing the two large-aggregating proteoglycans, CSPG2 and PGCA, and hyaluronic acid–binding link proteins HPLN1 and HPLN3. CSPG2 and PGCA are expressed in SMC, CSPG2 participates in the retention of lipoproteins, while PGCA is associated with plaque stability. Interestingly, in female patients, a reverse association was observed between estradiol levels and the content of CSPG2, PGCA and HPLN3, suggesting a potential role of estradiol in the formation of the plaque’s extracellular matrix [64].

Not only do proteomic profiles of atherosclerotic plaques differ between sexes, but the distribution of some circulating proteins in patients with CAD also shows marked sex-specific patterns [64, 65]. In the study by Liu et al. (2025), male and female patients with CAD were compared. As expected, several hormone-related proteins, including FSHB, INSL3, and EDDM3B, were differentially expressed between sexes. Moreover, 112 proteins were upregulated exclusively in females with CAD (but not in female controls), whereas 11 proteins were upregulated in males with CAD. The authors associated the upregulation of immune- and cytokine-mediated proteins in females with enhanced immune activation and potentially greater inflammatory contribution to plaque development in women. In contrast, higher expression of proteins involved in arginine metabolism and the endothelin signaling pathway in males may help explain the greater prevalence and earlier onset of CAD in younger men [65]. 

Advances in high-throughput plasma proteomics, analysed with machine learning (ML) techniques, showed promising results in improving the predictions of recurrent ASCVD events in secondary prevention [66], the prediction of all-cause mortality in patients at increased cardiovascular risk [67] and the prediction of ASCVDs incidence in healthy subjects. Such large-scale studies, with enough statistical power, hold the potential of providing significant information, which could ultimately lead to clinically relevant reclassification of ASCVD patients according to sex.

### Metabolomics

Metabolomics provides valuable insights into ASCVD by profiling the complex landscape of metabolites, end products of cellular processes that reflect real-time responses to genetic and environmental influences. As such, these metabolic signatures can be used to characterise both disease- and patient-specific phenotypes.

Recently, Couch et al. studied sex-associated metabolites in incident stroke, coronary heart disease, hypertension, and chronic kidney disease [68]. They included 2,114 participants from the REGARDS study and identified 51 metabolites associated with sex, including amino acids, acylcarnitines, bilirubin, bile acids, indolyl carboxylic acids, purines, sphingolipids, and glycerophospholipids in these patients. Higher levels of phosphatidylethanolamines, particularly phosphatidylethanolamine (34:2) and phosphatidylethanolamine (34:1), were linked to incident stroke only in women. Additionally, elevated uric acid was associated with hypertension exclusively in women, and lower betaine levels were seen only in women with CKD [68].

Using data from 1756 fasting serum samples of the KORA F4 population cohort, Krumsiek et al. observed significant differences between men and women in more than a third of 507 blood metabolites, with 77.2% of hits verified in an independent population cohort of 1000 subjects [69]. The study showed that in addition to the expected higher levels of androsterone and its derivatives and increased blood concentrations of amino acids, particularly branched-chain amino acids (BCAAs), were present in men [69]. These BCAAs, essential for muscle metabolism, showed significant gender differences in their basal levels and oxidation kinetics during exercise and are regarded as early markers for insulin resistance and diabetes, potentially impacting diabetes care. Other differences include gamma-glutamyl dipeptides, associated with the GGT enzyme, a marker for alcohol consumption and obesity, and a risk factor for CHD and overall mortality. Despite these metabolic differences, the same study [69] did not report on major gender-specific genetic influences on metabolite levels determined by gender-stratified GWAS, except for a single gene-metabolite association (CPS1-glycine). This indicates that sexual dimorphisms in gene expression are not primarily driven by genetic variants. On the other hand, sex differences in lipid profiles have been linked to X chromosome dosage. Women with Turner syndrome (monosomy 45X) have higher total cholesterol, LDL-C, and triglycerides compared to 46XX women with premature ovarian failure [70]. Men with Klinefelter syndrome (47XXY) exhibit higher rates of dyslipidemia and cardiometabolic risk factors [71]. Genetic variants on the X chromosome, often excluded from GWAS analyses, can contribute significantly to lipid metabolism. For instance, variants on chromosome Xq23 are associated with reduced total cholesterol, LDL-C, and triglycerides [72]. Although the Y chromosome has been linked to CVD risk factors and hypertension, a large study from the UK Biobank found no association between the male-specific Y chromosome region and lipid levels or cardiovascular risk [73]. Studies in diverse populations have revealed age-dependent differences in circulating metabolomic profiles between men and women. Bell et al. analyzed data from 7,727 offspring (49% male) and 6,500 parents (29% male) [74]. Their results indicate that from adolescence onwards, males have higher levels of VLDL triglycerides, while females exhibit higher levels of LDL cholesterol, apolipoprotein-B, and inflammatory glycoprotein acetyls, even when adjusted for BMI. These findings highlight that males consistently have higher triglyceride levels across life stages, while HDL cholesterol levels are lower in males post-puberty [74]. A recent study by Tabassum et al. observed age-sex interactions in as many as 121 lipid species, while 39 species displayed opposite age-related trends in men and women. For example, most phosphatidylcholines increased with age in women. In men, only phosphatidylcholines with polyunsaturated fatty acids changed with age, while those with saturated and monounsaturated fatty acids did not show significant age-related changes [75]. Beyene et al. [76] also reported higher levels of phosphatidylcholines, phosphatidylinositols, sphingomyelins, phosphatidylamines, ceramides, and triacylglycerides in postmenopausal women. Higher levels of lysophosphatidylcholines (LPCs) and ether-linked phospholipids in women might be due to elevated phospholipase A2 (PLA2) levels, an independent risk factor for CVD [76]. Triacylglycerides exhibited opposite age-related trends in men and women, being significantly higher in men until the age of 60 [75]. Dunn et al. found that eight DAGs were higher in relative concentration in women compared to men including DAG 44:6 and DAG 46:2 [77]. Most cholesteryl esters, ceramides, lysophospholipids, and glycerides were higher in men aged 45–50 years compared to women of the same age. However, these differences narrowed or even reversed with increasing age. Specifically, ceramide(d18:1/24:0) and ceramide(d18:1/24:1) were significantly higher in younger men compared to women, whereas in older men and women these differences were lost [75]. Similarly, Weir et al. [78] also found higher levels of ceramides (long chain ceramides Cer 22:0, Cer 24:0, and Cer 24:1) in men compared to women. The authors suggested this difference might be related to the differential expression of ceramide synthase 2 (CerS2), which produces ceramides using long chain fatty acids [78]. Beyene et al. observed generally higher ceramide levels in women, except for ceramides containing a 24:0 fatty-acyl chain, which were significantly higher in men. In line with these reports, recent findings have also revealed clear biological sex differences in sphingolipid ratios [79]. Men had distinct ceramide-to-S1P ratios compared with women, both under basal conditions and after estrogen treatment. Interestingly, although men had lower ceramide/S1P ratios – suggesting a more favourable lipid profile – endothelial cells from men produced less estrogen-induced H₂O₂, indicating sex-specific differences in redox regulation [79]. Acute exposure to estrogen promoted NO-mediated dilation in arterioles from healthy women via the NSmase/ceramide/S1P pathway, whereas this effect was significantly reduced in men. However, prolonged estrogen exposure altered endothelial signalling dynamics, increasing both ceramide and H₂O₂ production while impairing NO-mediated vasodilation [79]. Together, these results highlight a complex interplay between estrogen signalling and sphingolipid metabolism that may contribute to sex-specific differences in vascular function and CVD risk.

Cholesteryl esters and phosphatidylcholines with C20:3 and C20:4 fatty acids are increased with age in women but decreased in men, while those with C20:5 and C22:6 fatty acids are increased with age in both sexes [75]. A meta-analysis of 51 studies found significantly higher levels of long-chain polyunsaturated fatty acids (LCPUFA), arachidonic acid (ARA) and docosahexaenoic acid (DHA), in total plasma lipids and plasma phospholipids in women compared to men, while no gender difference was observed in their respective precursors, linoleic acid (LA) and alpha-linolenic acid (ALA) [80]. The synthesis of ARA and DHA from LA and ALA involves elongases and desaturases, which may differ in expression between sexes, potentially contributing to these differences. Furthermore, while estrogen is thought to affect LCPUFA levels, higher LCPUFA concentrations been observed even in postmenopausal women compared to men, indicating the observed differences may be independent of estrogen status [81]. Sphingomyelin (SM) levels were consistently higher in women and increased with age, consistent with the known age-related increase in cardiovascular disease (CVD) risk in middle-aged women [75]. Similarly, several studies showed that the strongest association was found between sex and SMs containing the d18:2 sphingoid base [76, 78]. This base, present in 21% of sphingolipids, contains an extra double bond introduced by fatty acid desaturase 3 (FADS3), an enzyme shown to have differential activity between men and women [76]. In addition, Verhaar et al. [82] found that SM 38:3, SM 42:4, SM 40:3, and SM 38:1 were significant predictors for both systolic blood pressure (BP) and heart rate variability (HRV) in men. Authors link these sphingolipids with the synthesis of ceramide, which suppresses phosphorylation of Akt and eNOS, potentially inducing vasoconstriction. They hypothesize that established sex differences in sphingolipid levels may be due to estrogen effects on adipose tissue or sphingomyelinase expression [82].

Although metabolomics offers potentially valuable information, large heterogeneity of data resulting from the use of different metabolomics platforms and methods, use of different sample types, different age stratification of cohorts, statistical approaches and population size significantly affect the results and prevent clear conclusions. Additional metabolome modulators associated with sex-differences observed in ASCVD patients range from existing metabolic conditions, diet, medications, gut microbiome, exercise and stress to lifestyle, behavioural, social and geographic factors [73, 82–86].

The fact is that sex is still insufficiently considered in metabolomics ASCVD research, despite its clinical potential for the personalisation of prevention, diagnosis and therapy. Studies have analysed the metabolome in atherosclerotic plaques [87, 88], and there is an ongoing need for spatial metabolomics to accurately determine the phenotype of plaques, predict their progression and optimise their treatment [89, 90]. However, these studies have not yet considered sex as a variable of interest, so there is a large research gap that needs to be filled in the coming years.

## Conclusions and Future Directions

Advances in omics technologies and molecular research now offer an unprecedented opportunity to uncover sex-specific mechanisms in ASCVD [91]. Leveraging these tools to improve understanding and management of ASCVD in women is not just important—it is an imperative. New insights should inform the development of sex-specific diagnostic tools and clinical guidelines to enhance the detection, prognosis, and management of adverse cardiovascular events in women.

An analysis of 771 preclinical articles on atherosclerosis and other vascular diseases published in leading American Heart Association journals from 2006 to 2016 revealed that fewer than 25% included both males and females [92]. This trend persisted even in 2017 and 2018 [93–95]. To advance the discovery of sex-specific mechanisms and biomarkers for ASCVD, it is essential that future studies and clinical trials ensure the inclusion of a substantial number of women representing diverse ages, ethnicities, and socioeconomic backgrounds. Although some large-scale multiomics studies aim to evaluate the risk of adverse CVD outcomes, many still neglect to conduct sex-specific analyses. Even when statistical power is limited, presenting sex-stratified data—at least in supplementary materials—should be considered a standard practice. These efforts have the potential to significantly enhance our understanding of sex-based differences, offering valuable insights and guiding future research directions.

In addition to ASCVD-specific challenges, integrating multiomics data and applying sequential AI and ML approaches introduces a distinct set of obstacles. The sheer volume of data generated by omics studies poses significant bottlenecks in storage, management, and computational capacity. Preprocessing and analyzing such datasets require extensive resources, including substantial storage space and high computational power, making large-scale analyses particularly demanding. The success of AI/ML-driven methods also hinges on the availability of robust, reusable datasets. For ML algorithms to be reliable for clinical application, rigorous validation is essential. However, the sensitive nature of omics data and strict legal frameworks, such as GDPR and various national regulations, often impede data sharing between institutions. This issue is further exacerbated by the absence of standardized protocols for data curation and sharing, limiting collaboration and slowing progress. One promising solution is the adoption of synthetic data, wherein patient information is modified to safeguard privacy while retaining its utility for ML modeling and validation. If widely implemented, this approach has the potential to transform data sharing in multiomics research and foster broader collaboration. Additionally, establishing international repositories with harmonized data-sharing agreements and secure access frameworks could address many of these challenges, facilitating more effective use of multiomics data.

Recent international initiatives, such as the MSCA SE CardioSCOPE and the COST Action AtheroNET, and NextGen have made significant strides in addressing these challenges. By fostering international collaboration, these projects aim to advance our understanding of sex differences in atherosclerosis, improve strategies for data sharing and reusability, and develop novel approaches to ASCVD management. Their focus on integrating multiomics with AI highlights the potential of these technologies to personalize prevention and treatment strategies.

## Key References

- Hartman RJG, Owsiany K, Ma L, Koplev S, Hao K, Slenders L, et al. Sex-Stratified Gene Regulatory Networks Reveal Female Key Driver Genes of Atherosclerosis Involved in Smooth Muscle Cell Phenotype Switching. Circulation. 2021;143:713–26. 10.1161/CIRCULATIONAHA.120.051231.
  - **○ **System-biology study uncovering female-specific gene regulatory networks in atherosclerotic tissue that identify key driver genes in smooth muscle cells, showing that in women these genes promote Klf4-dependent phenotypic switching of SMCs and thus may contribute to sex differences in plaque biology.
- Sukhavasi K, Mocci G, Ma L, Hodonsky CJ, Diez Benevante E, Muhl L, et al. Single-cell RNA sequencing reveals sex differences in the subcellular composition and associated gene-regulatory network activity of human carotid plaques. Nat Cardiovasc Res. Nature Publishing Group; 2025;4:412–32. 10.1038/s44161-025-00628-y.
  - **○ **Deep single-cell sequencing shows sex-specific plaque programs in carotid stenosis, with females enriched for osteogenic smooth muscle cells, immunomodulating macrophages and endothelial-to-mesenchymal transition, and males enriched for chondrocytic smooth muscle cells, tissue-remodeling macrophages and angiogenic endothelium. Integrating sex-biased cell states with gene-regulatory networks revealed male-specific GRN195 and female-specific GRN33/GRN122, with GRN195 functionally validated as a male endothelial therapeutic target.
- Diez Benavente E, Hartman RJG, Sakkers TR, Wesseling M, Sloots Y, Slenders L, et al. Atherosclerotic Plaque Epigenetic Age Acceleration Predicts a Poor Prognosis and Is Associated With Endothelial-to-Mesenchymal Transition in Humans. Arterioscler Thromb Vasc Biol. 2024;44:1419–31. 10.1161/ATVBAHA.123.320692.
  - **○ **Whole-genome methylation of carotid plaques and blood from the Athero-Express endarterectomy cohort was used to calculate epigenetic age acceleration (EAA), which emerged as a strong, independent marker of poor outcome in severe atherosclerosis. Plaque EAA was tied to mesenchymal endothelial and smooth muscle cells, validated endothelial-to-mesenchymal transition, and suggests epigenetic aging mechanisms as potential therapeutic targets.
- Almalki WH. Unraveling the role of Xist RNA in cardiovascular pathogenesis. Pathol Res Pract. 2024;253:154944. 10.1016/j.prp.2023.154944.
  - **○ **Review paper examining the role of Xist RNA in cardiovascular pathogenesis and emphasizing its emerging influence on sex-biased cellular responses in heart and vascular disease.
- Aragam KG, Jiang T, Goel A, Kanoni S, Wolford BN, Atri DS, et al. Discovery and systematic characterization of risk variants and genes for coronary artery disease in over a million participants. Nat Genet. Nature Publishing Group; 2022;54:1803–15. 10.1038/s41588-022-01233-6.
  - **○ **A large GWAS of coronary artery disease, identifying over 250 risk loci—including many newly discovered—and prioritizing likely causal variants and genes through fine-mapping, cross-ancestry analyses, and multiple integrative approaches. Experimental validation using CRISPR–Cas9 confirmed a functional enhancer in MYO9B, highlighting mechanisms involving vascular cell motility and other developmental and signaling pathways in CAD pathogenesis.
- Tcheandjieu C, Zhu X, Hilliard AT, Clarke SL, Napolioni V, Ma S, et al. Large-scale genome-wide association study of coronary artery disease in genetically diverse populations. Nat Med. 2022;28:1679–92. 10.1038/s41591-022-01891-3.
  - **○ **A large ancestrally diverse GWAS of coronary artery disease, identifying 95 novel loci (including nine on the X chromosome).
- Theofilatos K, Stojkovic S, Hasman M, van der Laan SW, Baig F, Barallobre-Barreiro J, et al. Proteomic Atlas of Atherosclerosis: The Contribution of Proteoglycans to Sex Differences, Plaque Phenotypes, and Outcomes. Circ Res. 2023;133:542–58. 10.1161/CIRCRESAHA.123.322590.
  - **○ **Comprehensive proteomic mapping of human atherosclerotic plaques revealing how proteoglycan-driven pathways contribute to sex differences, plaque phenotype variation, and clinical outcomes.
- Liu Y, Wang Z, Collins SP, Testani J, Safdar B. Sex differences in proteomics of cardiovascular disease – *Results from the Yale-CMD registry*. IJC Heart Vasc. 2025;58:101667. 10.1016/j.ijcha.2025.101667.
  - **○ **Plasma proteomics in the Yale-CMD registry revealing sex-specific protein signatures in coronary artery disease and microvascular dysfunction, with women showing upregulation of immune and metabolic pathways and men showing increased endothelial and angiogenesis-related proteins.
- Couch CA, Ament Z, Patki A, Kijpaisalratana N, Bhave V, Jones AC, et al. Sex-Associated Metabolites and Incident Stroke, Incident Coronary Heart Disease, Hypertension, and Chronic Kidney Disease in the REGARDS Cohort. J Am Heart Assoc. 2024;13:e032643. 10.1161/JAHA.123.032643.
  - **○ **Large-scale metabolomics study identifying sex-specific metabolic signatures associated with major cardiovascular outcomes, underscoring the value of metabolomics for understanding sex differences in disease risk.
- Tabassum R, Ruotsalainen S, Ottensmann L, Gerl MJ, Klose C, Tukiainen T, et al. Lipidome- and Genome-Wide Study to Understand Sex Differences in Circulatory Lipids. J Am Heart Assoc. 2022;11:e027103. 10.1161/JAHA.122.027103.
  - **○ **Comprehensive lipidomic and genomic investigation revealing biologically meaningful sex differences in lipid regulation; provides foundational evidence for sex-informed lipidomics research.
- SenthilKumar G, Zirgibel Z, Jaramillo-Torres MJ, Limpert RH, Cohen KE, Shult C, et al. Estrogen Influences Human Microvascular Endothelial Function Via Sex-Specific Regulation of Sphingolipids. JACC Basic Transl Sci [Internet]. American College of Cardiology Foundation; [cited 2025 Oct 31];0. 10.1016/j.jacbts.2025.101389.
  - **○ **Demonstrates a mechanistic link between estrogen signaling, sphingolipid regulation, and microvascular endothelial function, offering critical insight into sex-specific vascular biology.
- Sakkers TR, Mokry M, Civelek M, Erdmann J, Pasterkamp G, Diez Benavente E, et al. Sex differences in the genetic and molecular mechanisms of coronary artery disease. Atherosclerosis. 2023;384:117279. 10.1016/j.atherosclerosis.2023.117279.
  - **○ **Reviewing evidence from genetic and epigenetic studies pointing to sex-specific patterns in vascular remodeling, lipid metabolism, and endothelial function.

## Data Availability

No datasets were generated or analysed during the current study.
